# Downregulation of KIAA1199 by miR‐486‐5p suppresses tumorigenesis in lung cancer

**DOI:** 10.1002/cam4.3210

**Published:** 2020-06-09

**Authors:** Anqi Wang, Jianjie Zhu, Juan Li, Wenwen Du, Yang Zhang, Tingting Cai, Ting Liu, Yulong Fu, Yuanyuan Zeng, Zeyi Liu, Jian‐an Huang

**Affiliations:** ^1^ Department of Respiratory Medicine the First Affiliated Hospital of Soochow University Suzhou China; ^2^ Suzhou Key Laboratory for Respiratory Diseases Suzhou China; ^3^ Institute of Respiratory Diseases Soochow University Suzhou China

**Keywords:** cell motility, cell proliferation, KIAA1199, microRNA, miR‐486‐5p, Non‐small cell lung cancer (NSCLC)

## Abstract

Lung cancer is the primary cause of death among cancer patients in China, among which nonsmall cell lung cancer (NSCLC) makes up the great majority. Hence, it is imperative to identify the biomarkers and mechanisms involved in NSCLC oncogenesis. Our present research found that KIAA1199 expression was significantly increased in NSCLC and closely related to cell proliferation, motility, and poor prognosis. We demonstrated that knockdown of KIAA1199 reduced NSCLC cell growth and motility in vitro whereas overexpression of KIAA1199 had the opposite effect. Inhibition of KIAA1199 significantly suppressed tumor growth in mouse NSCLC xenograft models. Mechanistically, as an epidermal growth factor receptor (EGFR)‐binding protein, KIAA1199 promotes EGFR signaling and regulates EGFR‐dependent Src, Erk, and Akt phosphorylation, as well as downstream kinases in the EGF‐mediated EMT pathway. We demonstrated that KIAA1199 can function as a direct binding target for miR‐486‐5p and that miR‐486‐5p overexpression can attenuate proliferation and migration of NSCLC cells via regulating the EGFR signaling pathways. To conclude, our results defined KIAA1199 as an oncogenic protein that promotes cancer cell proliferation and migration by regulating EGF‐mediated signaling pathways. This study provided new insight into NSCLC oncogenesis, which could lead to the development of innovative therapeutic plans for NSCLC.

## Graphical abstract

In this study, we defined KIAA1199 as an oncogenic protein whose expression is markedly higher in lung cancer tissues and is regulated by miR‐486‐5p. KIAA1199 promotes cancer cell proliferation and migration by regulating EGFR‐mediated signaling pathways. These findings may lead to the development of novel therapeutic strategies for NSCLC.

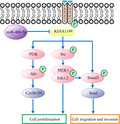

## 1 INTRODUCTION

The most common cancer on a global scale is lung cancer, while it is also the main reason for cancer‐related death.^1^, ^2^ Nonsmall‐cell lung cancer (NSCLC), mostly made up of adenocarcinoma or squamous cell carcinoma, accounts for more than 85% of lung cancers.^3^ Despite the development in advanced therapies, such as molecularly targeted drugs and antibodies, there has only been a minor increase of up to 18.1% in the projected 5‐year survival rate for NSCLC over the past several decades.^3^ NSCLC patients still face a medium survival of no longer than one year and a 5‐year survival rate beneath 20%.^4^, ^5^ Therefore, in‐depth research into NSCLC pathogenesis and new therapies with novel targets is urgently required.

Tumorigenesis involves a variety of signaling pathways and interrelated regulatory mechanisms, among which we have chosen to focus on the signaling pathways affecting tumor proliferation and motility. The Erk and PI3K‐Akt pathways are commonly dysregulated in malignant tumors including NSCLC,^6^ activated by epidermal growth factor receptor (EGFR) phosphorylation.^7^ These aberrantly activated pathways can lead to uncontrolled growth, cell proliferation, and reduced apoptosis.^8^, ^9^ On the other hand, epithelial‐mesenchymal transition (EMT) is regarded as a reversible process, where cells with epithelial phenotype are transferred into mesenchymal phenotype, and gains migratory and invasive properties during the process.^10^ It is also related to apoptosis resistance and extracellular matrix (ECM) production.^11^, ^12^, ^13^ In lung cancer, EMT is implicated with cell migration and invasion, development of cancer stem cells and increased resistance to apoptosis.^14^ In the present research, we studied oncogenesis by investigating the key components of the EGFR signaling pathways as well as EMT factors, such as Slug, Snail, N‐cadherin, and Vimentin. A better understanding of these tumorigenic signaling pathways is essential for the development of new treatments for NSCLC.

KIAA1199 was initially discovered as an inner‐ear gene and its mutation was responsible for nonsyndromic hearing loss.^15^ Many studies have reported that KIAA1199 is important in tumorigenesis. Upregulation of KIAA1199 has been defined to be related to tumor development and poor prognoses in multiple tumors, such as colorectal,^16^, ^17^ gastric,^18^, ^19^ breast,^20^, ^21^ and pancreatic^22^ cancers. Other research has already illustrated that KIAA1199 overexpression indicated poorer outcome, and promoted cell invasion and metastasis in lung cancer^23^ while missing a fully elucidated mechanism. Therefore, KIAA1199 was chosen for in‐depth molecular characterization. Here, we found that KIAA1199 promotes NSCLC growth and development by regulating EGFR‐mediated signaling pathways. Previous researches have shown that KIAA1199 can interact with EGFR and induce EGFR dephosphorylation, resulting in microtubule destabilization and increased cell motility.^24^, ^25^ KIAA1199 participates in EGF‐induced EMT as well as phosphorylation of downstream kinases, including Akt and Erk as mentioned above. In addition, data from the online database GEPIA2 (http://gepia.cancer‐pku.cn/) revealed a positive correlation between KIAA1199 and EGFR expression. Thus, KIAA1199 can be the key component connecting EGFR signaling to a variety of downstream pathways. Based on the above, we attempt to elucidate whether KIAA1199 can promote tumorigenesis of NSCLC by meditating EGFR‐induced signaling pathways in the present study.

The ectopic expression of microRNAs is associated with the development, progression, and prognosis of cancer.^26^ MicroRNAs are also related to the regulation of EMT during tumor progression,^27^ especially in the development of lung cancer.^28^, ^29^ MiR‐486‐5p, widely documented to be a tumor‐suppressive microRNA, has been recorded to suppress proliferation and invasion in many cancers.^30^, ^31^, ^32^ Our microRNA array results and GEO data analysis confirmed reduced miR‐486‐5p abundance in NSCLC tissues. Using online databases, we predicted that miR‐486‐5p can inhibit KIAA1199 expression via directly binding to the 3′‐UTR of KIAA1199. Here, we aimed to illustrate the interplay between miR‐486‐5p and KIAA1199 in NSCLC tumorigenesis.

Collectively, our research revealed that KIAA1199, negatively regulated by miR‐485‐5p, promotes oncogenesis and motility of NSCLC cells via EGFR‐mediated pathways. Hence, KIAA1199 could be a promising target to suppress cell proliferation and motility in NSCLC.

## 2 METHODS

### 2.1 Tissue samples

Paired NSCLC tissues and normal lung tissues (48 of each group) were collected between 2009 and 2013 from the First Affiliated Hospital of Soochow University. All chosen cases had been confirmed with NSCLC based on the International System for Staging Lung Cancer issued by International Association for the Study of Lung Cancer based on their histological and pathological characteristics. No patients had undergone any chemotherapy or radiotherapy before sample collecting. The collected tissue samples were then frozen at −80°C for further study. Each patient had agreed to an informed consent. The Ethics Committee of Soochow University had approved the study.

### 2.2 Cell culture

Human bronchial epithelial cell line BEAS‐2B and human NSCLC cell lines H226, A549, H1299, H1650, SPC‐A1, and H460 were obtained from the Cell Bank of the Chinese Academy of Science. Roswell Park Memorial Institute 1640 (RPMI 1640) or Dulbecco's Modified Eagle Medium (DMEM)/HIGH GLUCOSE medium (HyClone Logan, UT, USA) containing 10% fetal bovine serum (FBS) (Gibco), 100 U/mL penicillin, and 100 μg/mL streptomycin were used to culture cells. All the cells were grown at 37°C in a humidified atmosphere with 5% CO_2_.

### 2.3 RNA extraction, cDNA synthesis, and quantitative real‐time polymerase chain reaction (PCR) analysis

The RNAiso Plus kit (Takara, Kusatsu, Shiga, Japan) was applied for the extraction of total RNA from NSCLC cells. The isolated RNA was dissolved in diethylpyrocarbonate‐treated water and then reserved at −80°C for further usage. The M‐MLV First Strand kit (Life Technologies, Gaithersburg, MD, USA) was used to reverse‐transcribe RNA. Primers for KIAA1199, β‐actin, miR‐486‐5p, and U6 were purchased from Riobobio (Guangzhou, China). The reverse transcriptase reaction products were detected by quantitative real‐time polymerase chain reaction (qRT‐PCR) with 2x SYBR‐Green qPCR SuperMix (High ROX) (Bimake, Houston, USA) on the ABI StepOne Plus Real‐Time PCR system (Applied Biosystems) according to guidelines provided. All experiments were performed thrice. Ct values for KIAA1199 were normalized to internal control ACTB. Ct values for miR‐486‐5p were standardized to internal control U6. The primers sequences of KIAA1199 were 5′‐CCAGGAATGTTGAATGTCT‐3′ (forward) and 5′‐ATTGGCTCTTGGTGAATG‐3′ (reverse). Relative expression was calculated using the ΔΔC_t_ method.

### 2.4 Western blotting

RIPA buffer (Cell Signaling Technology) was used to isolate protein lysates from NSCLC cells. The extract was centrifugation at 12 000 rpm at 4°C for 15 minutes after 10 minutes of incubation and 10 minutes of vibration. The protein samples were then subpackaged and stored at −80°C. The sodium dodecyl sulfate polyacrylamide gel electrophoresis (SDS‐PAGE) was used to resolve the protein lysates. Subsequently, the samples were transferred to a nitrocellulose membrane (Millipore). After 2 hours of transferring, the membranes were immersed in 5% BSA in TBST (Tris buffer saline with Tween 20) for 1 hour and then immunoblotted with specific primary antibodies for more than 12 hours at 4°C. The bands were immunoblotted with matched secondary antibodies for 2 hours at 15°C to 25°C on the second day. An enhanced chemiluminescence (ECL) kit (Thermo Fisher) was used to visualize the bands. β‐actin protein levels were used to normalize sample loading. The antibodies used in the experiment were anti‐KIAA1199 (Santa Cruz, CA, USA), anti‐pEGFR (Tyr1068, 1H12), anti‐EGFR (D38B1), anti‐N‐Cadherin (13A9), anti‐pAKT (Ser473, D9E), anti‐pSmad3 (Ser423/425,C25A9), anti‐pERK (Thr202/Tyr204, D13.14.4E), anti‐AKT, anti‐ERK, anti‐Smad3, anti‐Slug, anti‐Snail, anti‐CyclinD1, anti‐β‐actin (all from Cell Signaling Technologies). The anti‐rabbit or anti‐mouse secondary antibodies were also from Cell Signaling Technologies, Dancers, MA, USA.

### 2.5 Establishment of KIAA1199‐knockdown and overexpression stable cell lines

In order to construct KIAA1199‐knockdown stable cell lines, a negative control: shNC sense 5′‐GGGTGAACTCACGTCAGAA‐3′, and two shRNAs were synthesized: shRNA‐1 5′‐GGTTATGACCCACCCACATAC‐3′, shRNA‐2 5′‐GGAGGCAAGCTGGTCATTAAAGACCACGA‐3′. The pGPU6/GFP/Neo vector (GenePharma, Shanghai, China) was used to load the mentioned sequences. Subsequently, the packaged lentiviruses were transduced into NSCLC cells. The transfected cells were filtered with 0.4ug/ml puromycin (Sigma‐Aldrich, St Louis, MO, USA) after cultured for 2 days. ShRNA‐1 was selected for further in vivo studies because it exhibited a higher KIAA1199 knockdown as examined on mRNA and protein level. The pCMV/AC/GFP vector (Genecopoeia, Guangzhou, China) was used to clone the full‐length KIAA1199 cDNA for overexpression experiments. NSCLC cells were transfected with pCMV/AC/GFP‐KIAA1199 vector or negative control. The cells were then selected with 1ug/ml Kanamycin to establish a stable cell line for the following experiments.

### 2.6 Luciferase reporter assay

We selected two potential miR‐486‐5p binding sites from online microRNA prediction databases to construct plasmids with KIAA1199 3′‐untranslated region (3′‐UTR) subcloned to the 3′‐end of a luciferase reporter downstream of the firefly luciferase gene. We synthesized a 1051‐bp sequence containing the projected miR‐486‐5p target sites and then clone it into the psi‐CHECK2‐control vector (Promega, Madison, WI, USA). Thus, we generated two psi‐CHECK‐KIAA1199‐3′‐UTR‐wild‐type and two psi‐CHECK2‐KIAA1199‐3′‐UTR‐mutant vectors. NSCLC cells were seeded onto 24‐well plates and co‐transfected with wild‐type or mutated plasmid and either miR‐NC or miR‐486‐5p mimics using Lipofectamine 2000 (Invitrogen). After incubation for 48 hours, the cell lysates were harvested. The firefly luciferase activity was assessed by the Dual‐Luciferase Reporter Assay Kit (Promega) and then standardized with renilla luciferase activity. Each experiment was performed thrice.

### 2.7 Cell proliferation analysis

Cell viability was assessed by the CCK‐8 assay and colony formation assay. 96‐well plates were seeded with 2 × 10^3^ transfected or control cells per well. The medium was changed and cell viability was assessed using Cell Counting Kit‐8 (Boster, Wuhan, China) at 24, 48, and 72 hours from seeding. A Thermo Scientific Varioskan Flash (Thermo Fisher) was applied to detect the absorbance values at 450 nm and 630 nm. Colony formation assay was performed by seeding 60‐mm plates with 300 transfected or control cells each plate. The cells were incubated for 7‐14 days, according to the growth rate. The colonies were counted after being fixed with methanol and stained. Each experiment was performed thrice.

### 2.8 Transwell assays

Transwell migration and invasion assays were conducted using 8.0 μm Transwell inserts (Corning, New York, NY, USA) with or without Matrigel (BD Science, Sparks, MD, USA). 4 × 10^4^ cells diluted in 200 μL RPMI‐1640 with 1% FBS were seeded onto the Transwell inserts. 800 μL RPMI‐1640 medium with 10% FBS filled the lower chamber as an attractant. The Transwell system was then placed at 37°C for 24 hours. After washed with PBS twice, the Transwell inserts were fixed with methanol for 30 minutes and then air‐dried. The cells migrated to the lower surface were stained with 0.1% crystal violet overnight afterward. Finally, the inserts were photographed and counted using a microscope (CKX41, Olympus, Japan). The Transwell inserts must be coated with diluted Matrigel and incubated at 37°C for 2 hours before invasion assay. The subsequent steps were similar to the former migration assay. Each experiment was performed thrice.

### 2.9 Wound healing assay

To assess their migration ability, cells were cultured in 6‐well plates. The cells were incubated at 37°C until a monolayer was formed and then scratched in straight lines with a new pipette tip. Subsequently, sterile PBS was used to wash cells and then we refilled the wells with complete medium. Photos were captured under a microscope (CKX41, Olympus) after an additional 24 hour. Each experiment was performed thrice.

### 2.10 Animal experiments

The Guide for the Care and Use of Experimental Animals Center of Soochow University was complied with throughout all the animal experiments conducted. We purchased female BALB/c athymic nude mice from the Experimental Animal Center of Soochow University. The mice were aged 4‐6 weeks, weighed 16‐20 g, and raised under specific pathogen‐free conditions. 150 μL A549 cell suspension (3 × 10^6^ cells) was inoculated into the front flank of nude mice to build the NSCLC xenograft model. The mice were randomly allocated into KIAA1199 knockdown group (n = 10) and control group (n = 10). Tumor volume and weight were carefully monitored every other day. In addition, we applied the formula $V=\left(L\times W^{2}\right)\times 0.5$ into calculating all tumor volume (*V*) each time, where length (*L*) and width (*W*) represent the longest and shortest diameters of the tumor.

### 2.11 Statistical analysis

GraphPad Prism 7.0 (GraphPad) was used for all statistical analyses. Statistical significance between two groups was performed by the two‐tailed Student's t‐test. SPSS 16.0 software (SPSS) and the Kruskal‐Wallis test were used to analyze clinicopathologic characteristics in the NSCLC samples. All experiments in this study were performed at least thrice. All numerical data are presented as means ± SEM *P* < .05 was considered statistically significant.

## 3 RESULTS

### 3.1 KIAA1199 is overexpressed in NSCLC tissue and cell lines

**Figure 1:**
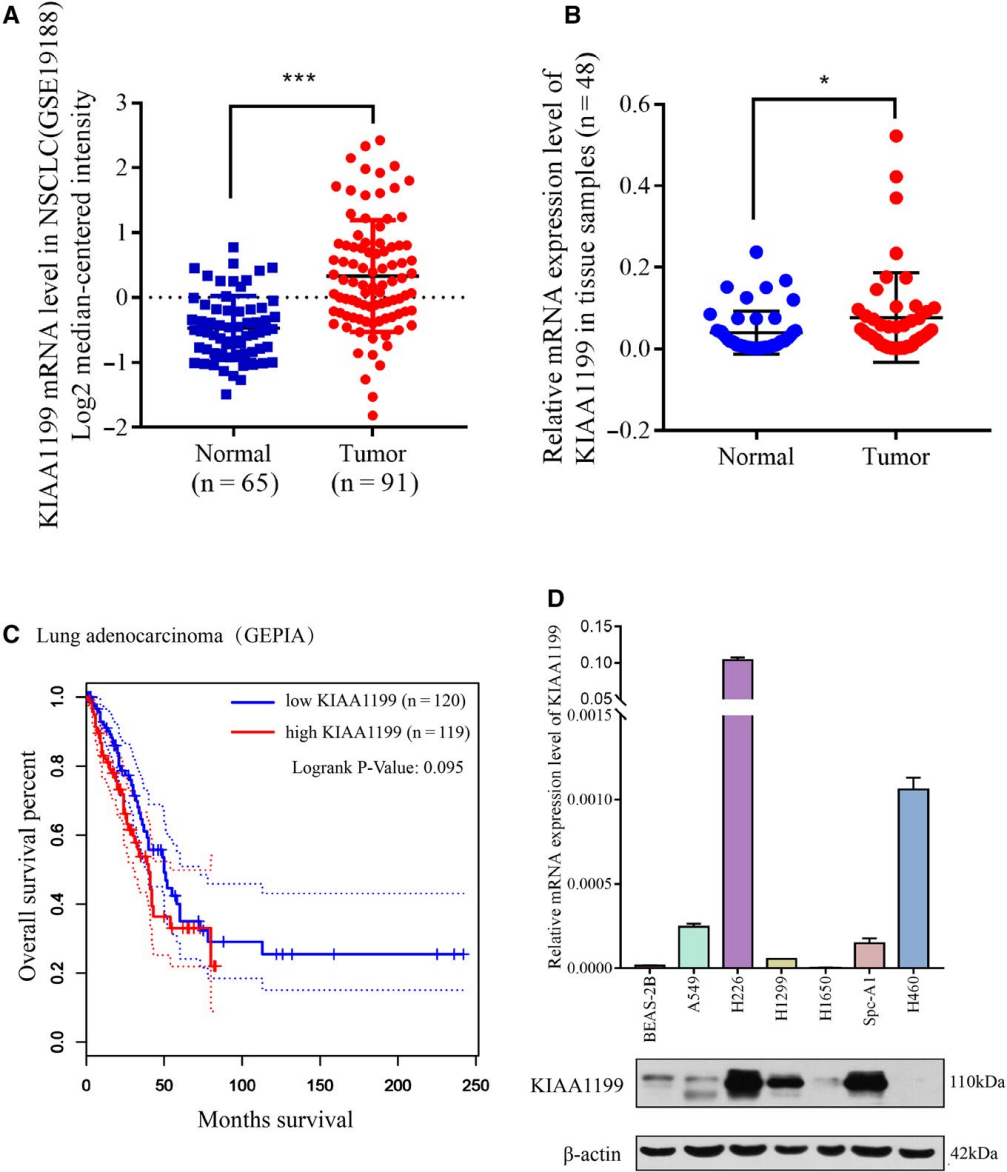
High expression levels of KIAA1199 in NSCLC tissues and cell lines. A, Statistics from a public database (GSE19188) revealed that KIAA1199 mRNA levels are significantly increased in NSCLC tissues in comparison to normal lung tissue samples. B, KIAA1199 mRNA levels were significantly increased in NSCLC tissues in comparison to adjacent noncancerous tissues that we collected. C, The relation of KIAA1199 and overall survival in 239 lung adenocarcinoma patients in the GEPIA2 database (http://gepia.cancer‐pku.cn/). D, The expression levels of KIAA1199 in six NSCLC cell lines and a bronchial epithelial cell line were examined. *P < .05, ***P < .001

Based on online public databases, we detected several differentially expressed genes in lung cancer, among which KIAA1199 caught our attention. Accumulating studies have demonstrated that KIAA1199 has emerged as a cell migration‐inducing protein in driving tumor progression. First, we examined KIAA1199 mRNA level in 91 NSCLC tissues and 65 noncancerous tissues from a public database (Gene Expression Omnibus, GSE19188). The results indicated that human NSCLC tissues contained a higher KIAA1199 mRNA expression level (Figure 1A). Secondly, the KIAA1199 mRNA levels in 48 paired NSCLC and adjacent noncancerous lung tissues were detected by qRT‐PCR analysis. We confirmed higher KIAA1199 expression in NSCLC samples than that in noncancerous tissues (Figure 1B), whereas KIAA1199 mRNA levels showed no statistical difference when we categorized all cases based on the demographic and clinical characteristics (Table 1). Additionally, KIAA1199 expression in seven cell lines indicated that KIAA1199 was upregulated in NSCLC cell lines on both mRNA and protein levels (Figure 1D). Furthermore, we evaluated the relativity between KIAA1199 expression and NSCLC prognosis by GEPIA2 database (http://gepia.cancer‐pku.cn/). The results suggested that KIAA1199 is prognostic of lower overall survival in NSCLC (Figure 1C). These observations illustrated upregulated KIAA1199 in NSCLC tissues and cell lines, implying poor prognosis. Therefore, KIAA1199 might be an important factor in NSCLC occurrence and progression.

**Table 1:** Demographic and clinical characteristics and levels of KIAA1199 mRNA expression in NSCLC tissue

Characteristics	Number of cases (%)	KIAA1199 mRNA expression	*P* value
Age			
≥66	20(41.7%)	0.1431 ± 0.0794	.489
<66	28(58.3%)	0.0845 ± 0.0251	
Gender			
Male	26(54.2%)	0.1312 ± 0.0620	.481
Female	22(45.8%)	0.0826 ± 0.2856	
Histological features			
Adenocarcinoma	30(62.5%)	0.1137 ± 0.0545	.947
Squamous cell carcinoma	8(16.7%)	0.1090 ± 0.0429	
Others	10(20.8%)	0.0945 ± 0.0487	
Tumor invasion depth			
T1, T2	40(83.3%)	0.1221 ± 0.0427	.102
T3, T4	8(16.7%)	0.0432 ± 0.0202	
Lymph node metastasis			
Yes	16(33.3%)	0.0834 ± 0.0314	.531
No	32(66.7%)	0.1216 ± 0.0517	
Distant metastasis			
Yes	5(10.4%)	0.0547 ± 0.0328	.255
No	43(89.6%)	0.1152 ± 0.0399	
Clinical stage			
I + II	31(64.6%)	0.1402 ± 0.0546	.125
III + IV	17(35.4%)	0.0519 ± 0.0131	
Degree of differential			
Low	24(50.0%)	0.1510 ± 0.0691	.250
Middle	24(50.0%)	0.0668 ± 0.0184	
Smoker			
Yes	19(39.6%)	0.1448 ± 0.0584	.138
no	29(60.4%)	0.0541 ± 0.0114	

Note

Data are presented as means ± SEM values. Unpaired t‐test was used for comparison between two groups, and Kruskal‐Wallis test was used for comparison between three or more groups.

### 3.2 Downregulation of KIAA1199 inhibits cell growth, migration, and invasion of NSCLC cells

**Figure 2:**
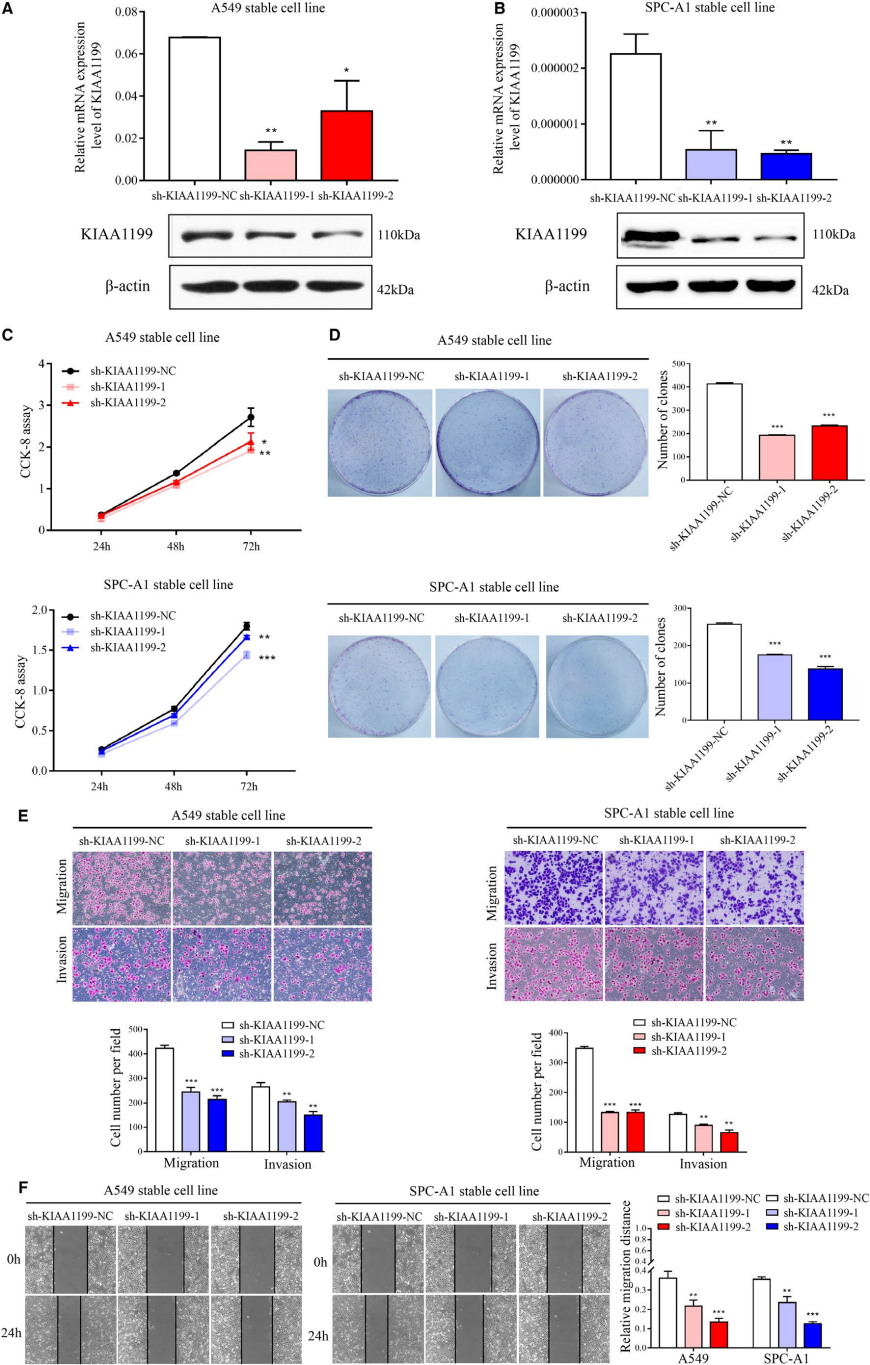
Inhibition of NSCLC cell pathogenesis by KIAA1199 knockdown. A, and B, KIAA1199 mRNA and protein levels in KIAA1199‐knockdown cell lines. C, The cell proliferation of KIAA1199‐knockdown cells was assessed by CCK‐8 assay. D, The characteristic images of the cell colony formation were captured. The colonies were quantified in the graph on the right. E, The migration and invasion abilities were inhibited in KIAA1199‐knockdown cells. F, Wound closure was delayed in KIAA1199‐silenced cells compared with control cells in the wound healing assay. Each experiment was performed in triplicate. Significant differences: *P < .05, **P < .01, ***P < .001

To elucidate the influence of KIAA1199 on NSCLC cell phenotype, we chose A549 and SPC‐A1 cell lines to construct stable KIAA1199‐knockdown cell lines. Both KIAA1199 mRNA and protein levels were markedly decreased in A549 and SPC‐A1 cells (Figure 2A,B). Next, we verified the influence of KIAA1199 on cell growth and motility of NSCLC cells. CCK‐8 assay showed obviously inhibited cell viability after silencing KIAA1199 expression (Figure 2C). A clonogenic assay has further confirmed that downregulation of KIAA1199 suppresses cell proliferation (Figure 2D). Moreover, the Transwell migration and invasion assays elucidated that suppression of KIAA1199 significantly attenuated the migratory and invasive ability of NSCLC cells (Figure 2E). The KIAA1199‐knockdown cells migrated into the scratch at a much slower speed than negative control cells in a wound‐healing assay, further validating that knockdown of KIAA1199 suppressed the migratory capacity of NSCLC cells (Figure 2F). These findings strongly indicated that the downregulation of KIAA1199 can inhibit cell growth and motility of NSCLC cells in vitro.

### 3.3 Upregulation of KIAA1199 promotes cell growth, migration, and invasion of NSCLC cells

**Figure 3:**
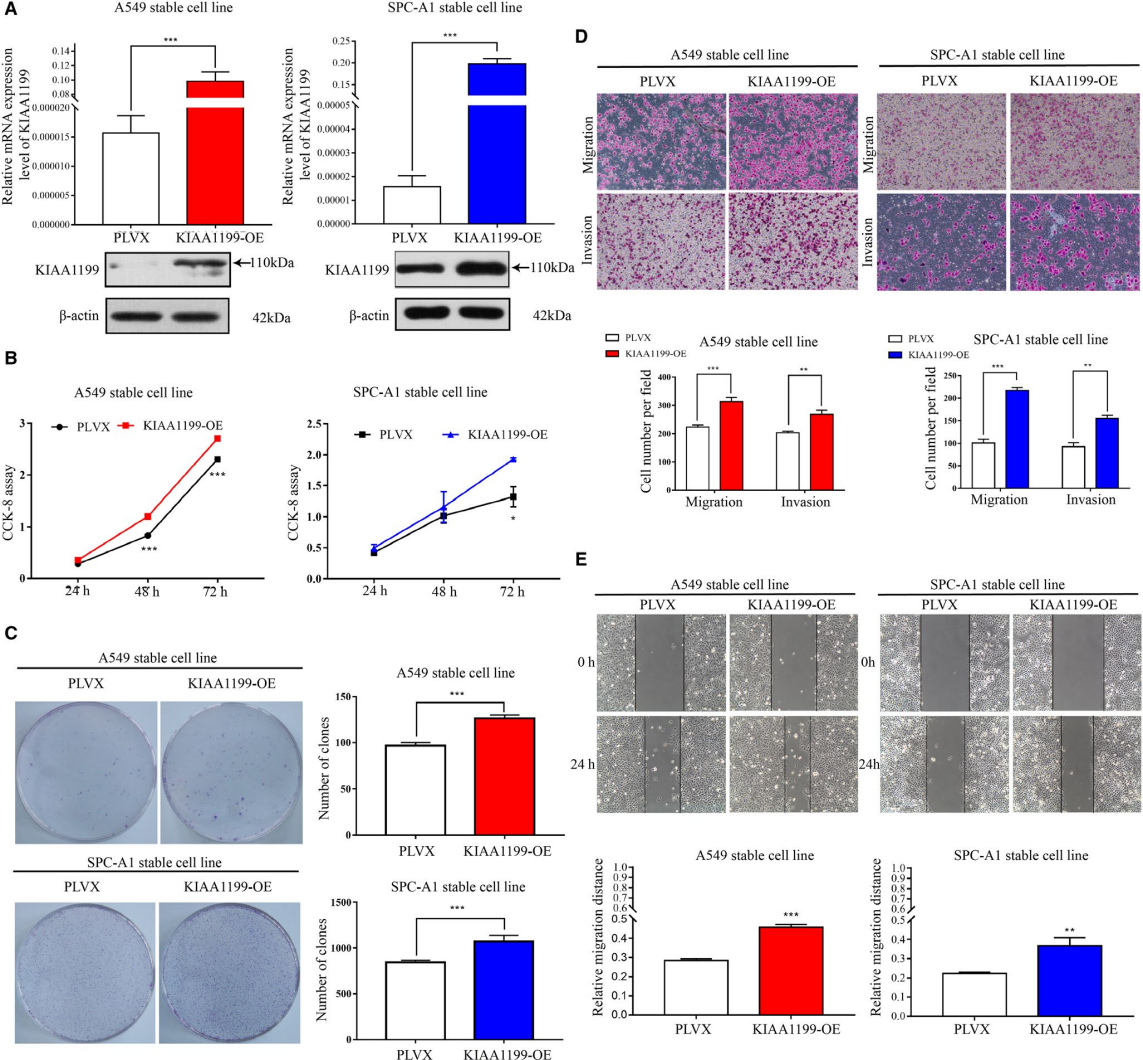
Promotion of NSCLC cell pathogenesis by KIAA1199 overexpression. A, KIAA1199 mRNA and protein levels in KIAA1199‐overexpressed cell lines. B, The cell proliferation of KIAA1199‐overexpressed cells was detected by CCK‐8 assay. C, The characteristic images of the cell colony formation were captured. The colonies were quantified in the graph on the right. D, Upregulation of KIAA1199 enhanced migration and invasion ability of NSCLC cells. E KIAA1199‐overexpressing cells migrated into the scratched wound faster than negative control cells in the wound healing assay. Each experiment was performed in triplicate. Significant differences: *P < .05, **P < .01, ***P < .001

To sequentially validate the function of KIAA1199, KIAA1199‐overexpression cell lines were established and confirmed based on the high KIAA1199 mRNA and protein levels (Figure 3A). Overexpression of KIAA1199 significantly increased cell proliferation compared to negative control cells, as examined by the CCK‐8 and clonogenic assays (Figure 3B,C). Additionally, we performed the transwell assay and wound healing assays to assess the influence of KIAA1199 overexpression on migratory and invasive capabilities of NSCLC cells (Figure 3D,E). Collectively, these findings suggested that KIAA1199 can strengthen cell growth and motility of NSCLC cells in vitro, implying that KIAA1199 may have a pro‐oncogenic role in NSCLC.

### 3.4 KIAA1199 improves the proliferation and motility of NSCLC cells via the EGFR signaling pathway

**Figure 4:**
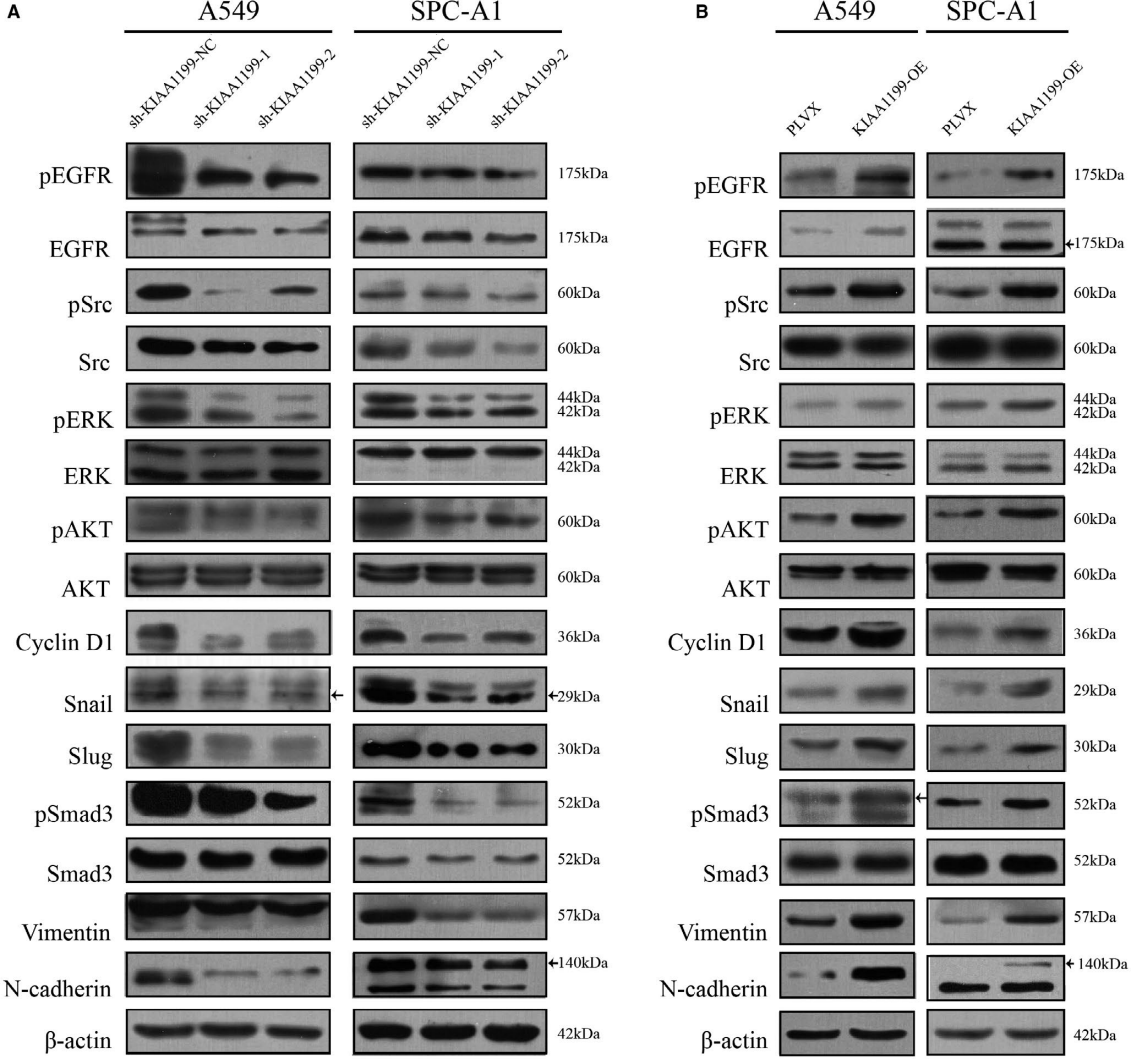
KIAA1199 is associated with EGFR signaling. A, Related signaling molecules were detected. The pEGFR, EGFR, pSrc, pErk, pAKT, pSmad3, and other EMT markers levels were significantly decreased in KIAA1199‐knockdown cells. B, In KIAA1199‐overexpressing cells, the expression of pEGFR, EGFR, pSrc, pErk, pAKT, pSmad3, and other EMT markers was increased compared to control cells

As seen above, KIAA1199 can promote cell proliferation and motility in NSCLC cells, while the underlying mechanism remained unclear. Previous research has shown that KIAA1199 was able to stabilize the EGFR protein and facilitate EGFR phosphorylation to promote tumor survival and migration.^25^, ^33^ Therefore, we detected the influence of KIAA1199 knockdown and overexpression on the pEGFR and EGFR levels as well as several proteins downstream EGFR signaling. As shown in Figure 4A, we found that pEGFR, EGFR, pSrc, pErk, pAKT, pSmad3, and other proteins involved in EGFR signaling were significantly reduced in KIAA1199‐knockdown cells as analyzed by western blotting, and opposite results were obtained in the KIAA1199 overexpression cell lines (Figure 4B). Moreover, KIAA1199 was reported to accelerate cell migration by aiding EGFR‐induced EMT.^33^ We next examined the changes in EMT‐related markers and verified that knockdown of KIAA1199 reduced the expression of transcription factors like Slug and Snail, as well as mesenchymal markers Vimentin and N‐Cadherin. On the contrary, upregulation of KIAA1199 increased the levels of Snail, Slug, N‐cadherin, and Vimentin. Combined with the above findings, these results suggested that KIAA1199 can mediate NSCLC cell proliferation and motility by modulating EGFR signaling.

### 3.5 KIAA1199 knockdown in NSCLC cells inhibits tumor growth in nude mice

**Figure 5:**
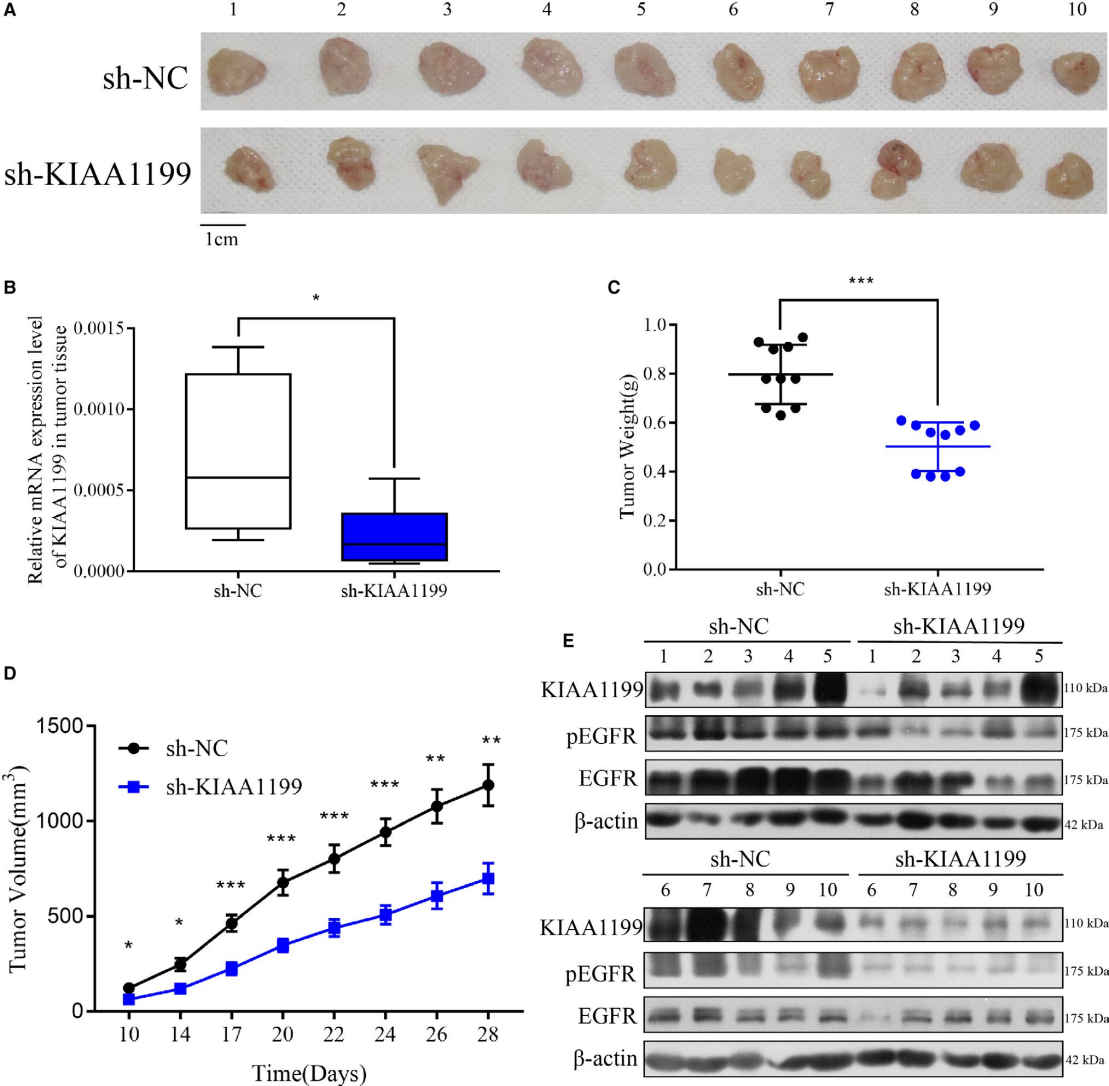
KIAA1199 accelerates tumor growth in vivo. A, KIAA1199‐knockdown NSCLC cell xenografts formed in nude mice (n = 10). By the end of the experiment, the xenograft tumors were dissected and photos of which were captured. B, A qRT‐PCR analysis was applied to examine the KIAA1199 mRNA levels in tumor tissues. C, Each tumor was weighed. D, The tumors were measured every other day and tumor growth curves were drawn for each group. E, Western blot analysis was applied to assess the protein expression in tumor tissues. KIAA1199, pEGFR, and EGFR levels were decreased in KIAA1199‐knockdown xenograft tumors than negative control one. Significant differences: *P < .05, **P < .01, ***P < .001

In order to further verify the role of KIAA1199 in mediating NSCLC cell proliferation in vivo, a subcutaneous mice model was established using negative control and KIAA1199‐knockdown stable cell lines in nude mice (BALB/c), which mimics the growth of NSCLC better than other animal models (Figure 5A). We observed that mice implanted with KIAA1199‐knockdown cells exhibited inhibited cell growth. We inoculated negative control cells and corresponding KIAA1199‐knockdown cells into BALB/C athymic mice. As expected, xenograft tumors generated by the KIAA1199‐knockdown cells were smaller and lighter than the negative control group (Figure 5C,D). The KIAA1199 mRNA expression of the KIAA1199‐knockdown group was significantly inhibited than the negative control group (Figure 5B). In addition, the protein electrophoresis assay revealed that the KIAA1199 and p‐EGFR levels were lower in the KIAA1199‐knockdown group (Figure 5E). These results suggested that reduced KIAA1199 expression can regulate EGFR signaling in vivo.

### 3.6 MiR‐486‐5p is downregulated in NSCLC and directly targets KIAA1199

**Figure 6:**
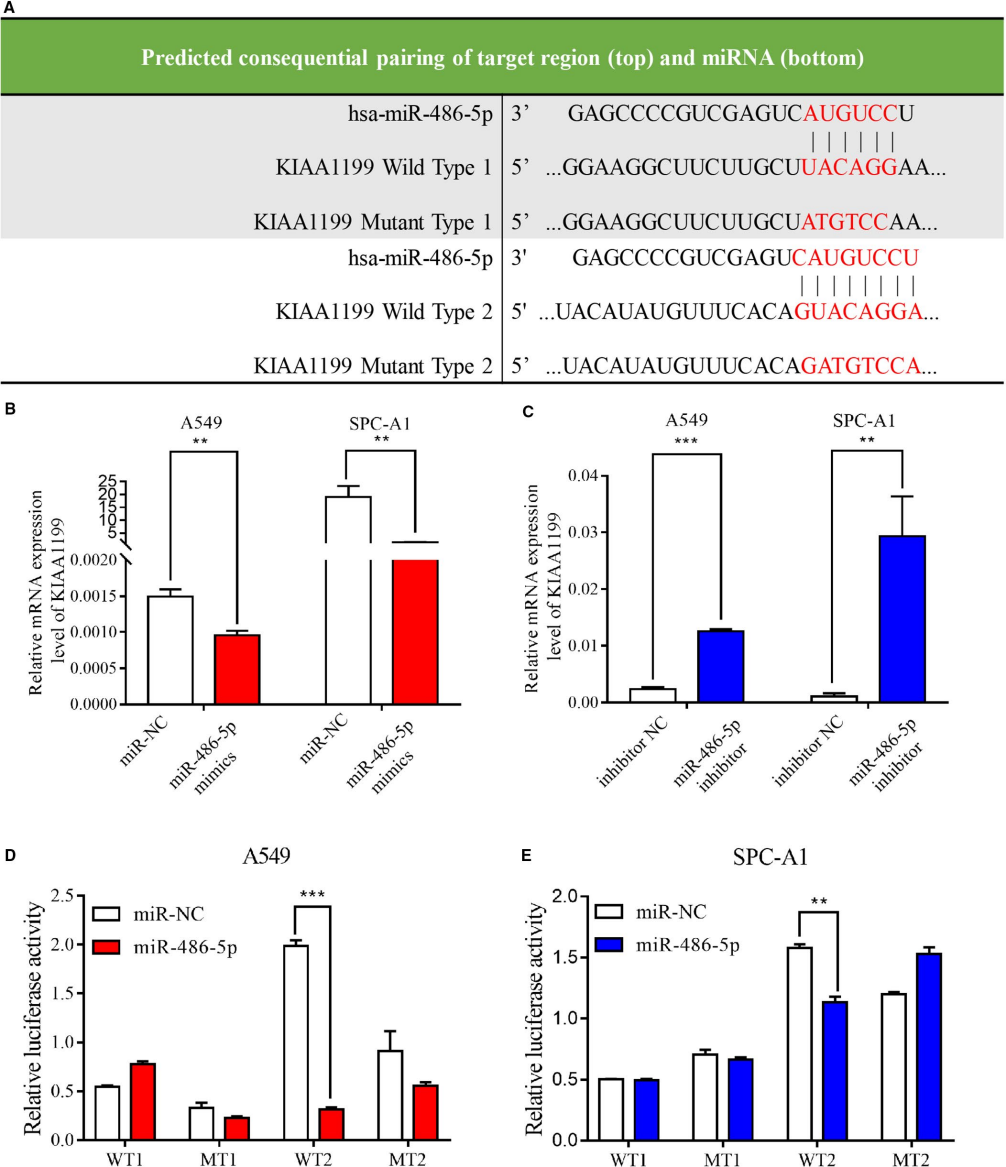
Regulation of KIAA1199 expression by miR‐486‐5p. A, The table showed the predicted miR‐486‐5p binding sites at position 895‐901 and 2374‐2385 of KIAA1199 3′‐UTR. B, The mRNA levels of KIAA1199 were lower in miR‐486‐5p‐upregulated cells. C, The mRNA levels of KIAA1199 were higher in miR‐486‐5p‐inhibited cells than control cells. D, and E, NSCLC cells were co‐transfected with wild‐type or mutant recombinant plasmid and negative control or miR‐486‐5p mimics. Relative luciferase activity was detected 48 hours afterward. Each experiment was performed in triplicate. Significant differences: **P < .01, ***P < .001

**Figure 7:**
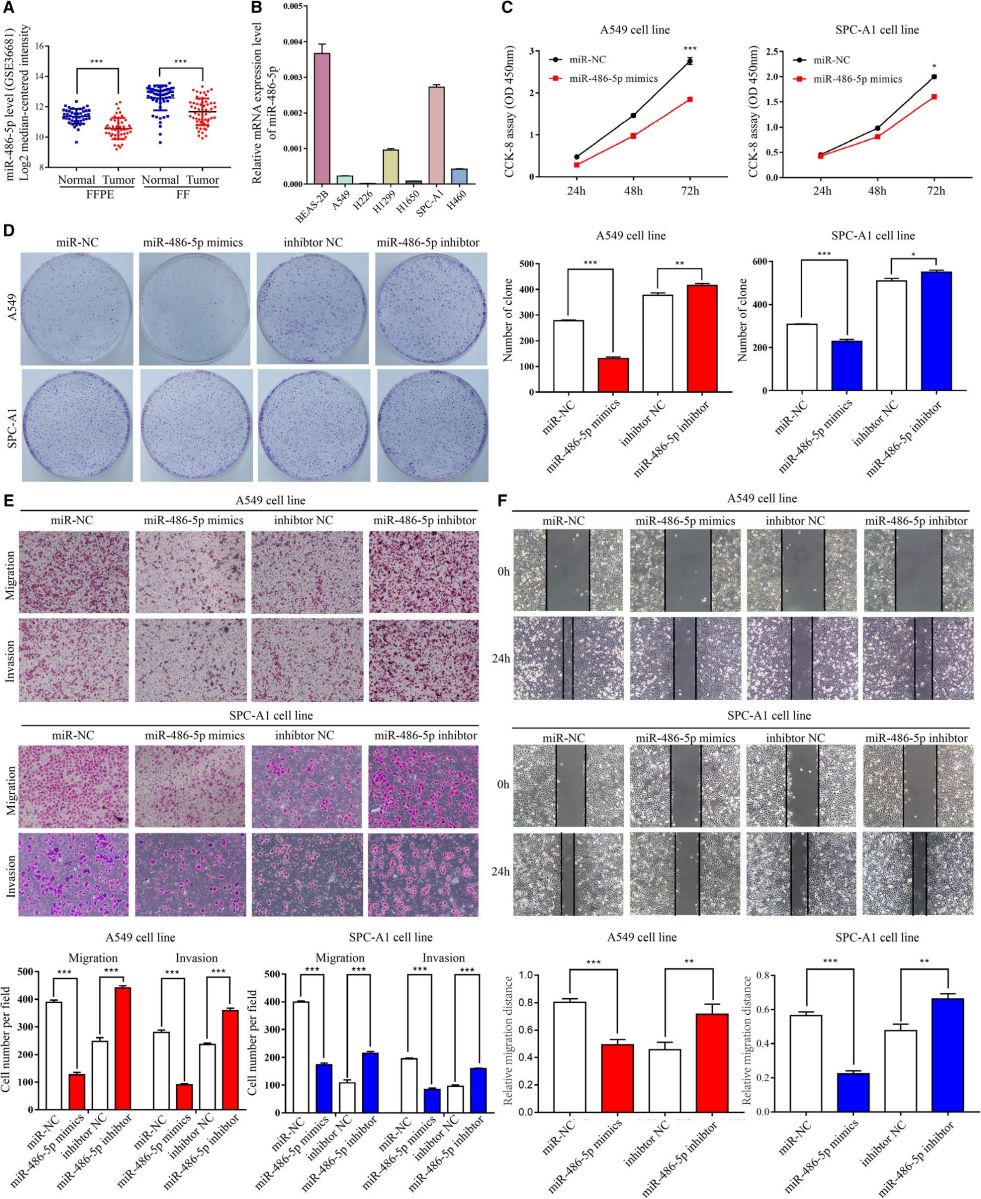
The expression and function of miR‐486‐5p in NSCLC. A, Statistics from Gene Expression Omnibus dataset (GSE36681) revealed that miR‐486‐5p mRNA levels were suppressed in NSCLC tissues. B, The mRNA levels of miR‐486‐5p in six NSCLC cell lines and a normal bronchial epithelial cell line. C, The cell proliferation of miR‐486‐5p‐upregulated and negative control cells was detected by a CCK‐8 assay. D, The representative images of the colonies generated by NSCLC cells were captured. The colonies were quantified in the graph on the right. E, Upregulation of miR‐486‐5p inhibited migration and invasion of NSCLC cells, whereas knockdown of miR‐486‐5p did the opposite. F, MiR‐486‐5p‐upregulated cells moved into the scratch more slowly while miR‐486‐5p‐downregulated cells were faster in the wound‐healing assay. Each experiment was performed in triplicate. Significant differences: *P < .05, **P < .01, ***P < .001

**Figure 8:**
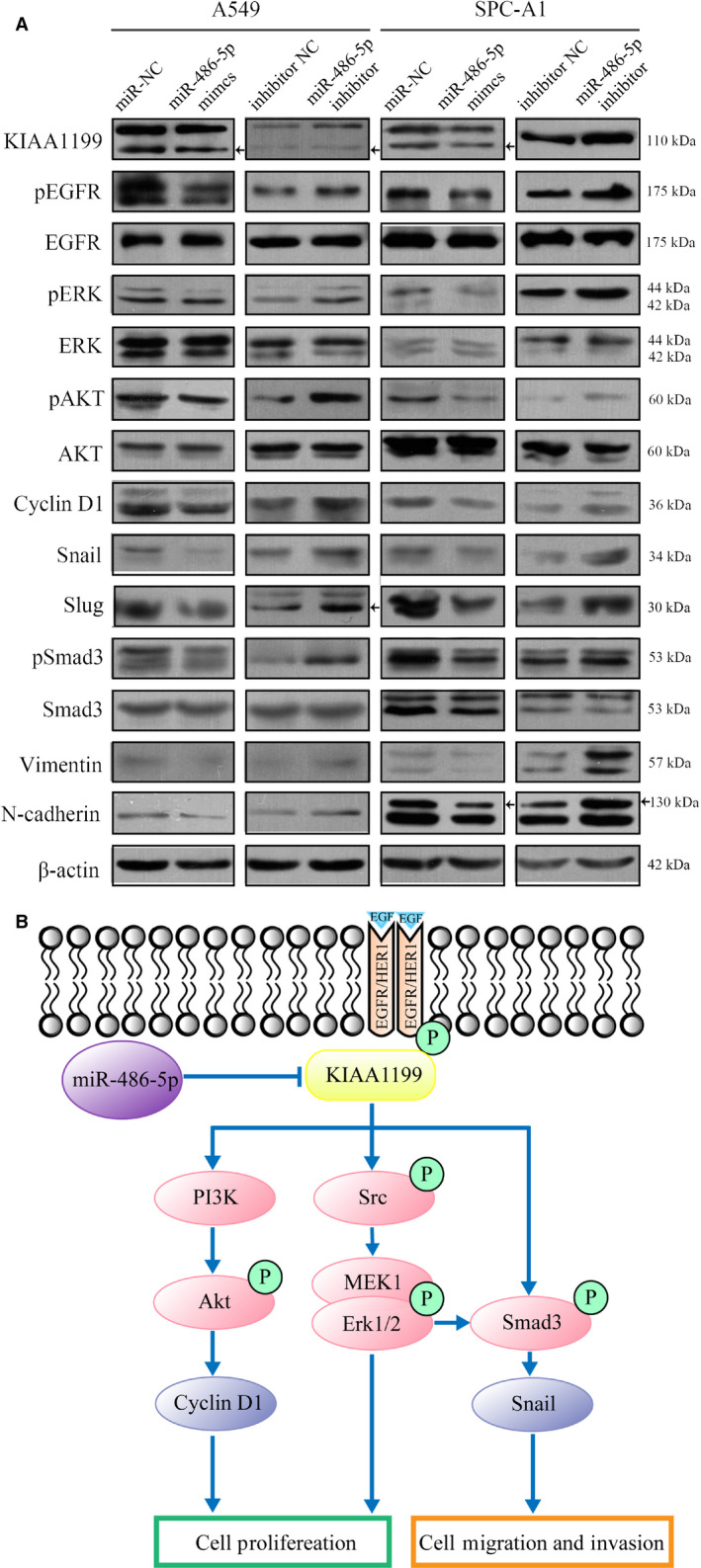
A, A549 and SPC‐A1 cells were transfected with miR‐486‐5p mimics or inhibitor for 72 hours. The levels of pEGFR, EGFR, pErk, Erk, pAKT, AKT, pSmad3, Smad3, and other EMT markers were analyzed by western blotting. B, A diagram shows the mechanism of which miR‐485‐5p interacts with KIAA1199 and their control of EGFR‐related signaling pathways

Since the expression regulation mechanism of KIAA1199 remains unknown and that miRNAs can directly regulate gene expression, we aimed to seek miRNAs that could target KIAA1199 in this study. To screen for upstream miRNAs that directly target KIAA1199, we searched online biological databases (TargetScanHuman: http://www.targetscan.org/ and miRanda: http://www.microrna.org/microrna/) and identified two putative binding sites for miR‐486‐5p on the 3′‐UTR of KIAA1199 mRNA (Figure 6A). We compared miR‐486‐5p mRNA levels of 45 Formalin‐fixed Paraffin‐embedded (FFPE) NSCLC tissues to 47 FFPE normal lung tissues, and 58 fresh‐frozen NSCLC tissues to 56 noncancerous lung tissues from public database (GSE36681). These findings revealed that miR‐486‐5p level was higher in noncancerous tissues than NSCLC tissues (Figure 7A). We quantified levels of miR‐486‐5p in NSCLC and human normal bronchial epithelial cells and found it to be downregulated in NSCLC cells (Figure 7B). Thus, it is highly possible that KIAA1199 expression can be negatively regulated by miR‐486‐5p. To experimentally validate that KIAA1199 expression is regulated by miR‐486‐5p, we transfected NSCLC cells with miR‐486‐5p mimics or inhibitor respectively to upregulate or downregulate miR‐486‐5p expression. As expected, miR‐486‐5p overexpression significantly suppressed the mRNA and protein expression of KIAA1199 (Figures 6B and 8A). Conversely, miR‐486‐5p inhibition increased the mRNA and protein levels of KIAA1199 (Figures 6C and 8A). Consequently, we chose miR‐486‐5p as the candidate miRNA for further investigation. For luciferase reporter assays, two wild‐type luciferase reporter plasmids were constructed by cloning the projected miR‐486‐5p binding sites into KIAA1199 3′‐UTR, downstream of the firefly luciferase gene. Whereas mutated fragments were subcloned into the vector to synthesize mutant plasmids. The findings indicated that the bond between miR‐486‐5p mimics and KIAA1199 predicted that binding site 2 inhibited luciferase activity in wild type 2 cells. Conversely, mutation of the binding site 2 abolished the inhibiting effect induced by miR‐486‐5p. The experiment in WT1 cells and MT1 cells showed no significant difference in luciferase activity (Figure 6D,E). Hence, predicted binding site 2 was verified to be valid between the two sites. In conclusion, these results validated that miR‐486‐5p directly targets KIAA1199 and negatively regulates it.

### 3.7 MiR‐486‐5p overexpression inhibits NSCLC cell proliferation and motility through EGFR signaling

To confirm the effects of miR‐486‐5p in NSCLC cells, we used miR‐486‐5p mimics and inhibitor to overexpress or silence miR‐486‐5p in A549 and SPC‐A1 cells. In a CCK‐8 assay, miR‐486‐5p overexpression in NSCLC cells significantly attenuated proliferation rate (Figure 7C). The colony formation showed similar results (Figure 7D). A Transwell assay verified that miR‐486‐5p significantly attenuated the migration and invasiveness of NSCLC cells (Figure 7E). Cells overexpressing miR‐486‐5p migrated into the scratch at a much slower speed than control cells in the wound healing assay (Figure 7F). From the information above, we concluded that miR‐486‐5p inhibited NSCLC cell proliferation and motility. Mechanistically, when compared to control group, pEGFR, EGFR, pAKT, pErk, pSmad3, and EMT markers expression were evidently decreased in cells transfected with miR‐486‐5p mimics (Figure 8A), which is in line with the results obtained from the KIAA1199‐knockdown cells. The converse was observed in miR‐486‐5p‐inhibited cells (Figure 8A), which further confirmed our hypothesis that miR‐486‐5p regulates KIAA1199 to stabilize EGFR and mediate downstream signaling (Figure 8B).

## 4 DISCUSSION

In this study, the characterization of KIAA1199 was described as an oncogenic protein whose expression is markedly higher in lung cancer tissues and is mediated by miR‐486‐5p. While attenuating KIAA1199 can suppress NSCLC cell proliferation and migration, it also downregulates the expression of several transcription factors involved in EMT process and EGFR signaling. Finally, we demonstrated here that KIAA1199 promotes NSCLC oncogenesis through EGFR signaling, which agreed with previous findings in other cancer types.

Cancer is a malignant tumor characterized by unlimited growth potential which expands locally by invasion and systemically by metastasis. While indefinite growth and invasion are critical hallmarks of cancer, the exact mechanism remains to be fully elucidated,^34^ especially in lung cancer which we have focused on. We demonstrated here that KIAA1199 mRNA and protein levels are aberrantly increased in NSCLC tissues in comparison to normal tissues. Consistent with previous research, we found that upregulated levels of KIAA1199 could lead to tumor progression, invasion, and poor prognosis.

KIAA1199 was initially identified as a long cDNA in the Human Unidentified Gene Encoded (HUGE) protein database,^34^ the mutation of which can lead to nonsyndromic hearing loss.^35^ Nowadays, KIAA1199 has also been known as cell migration‐inducing protein (CEMIP)^36^ as several pieces of research have indicated that KIAA1199 is involved in proliferation and migration of tumor cells, and poor prognosis in different cancers.^37^, ^38^ Although the influence of KIAA1199 in tumor progression has been reported in gastric,^18^, ^39^ colon,^16^, ^17^ pancreatic,^22^ and cervical cancer,^24^ the specific molecular mechanisms in lung cancer remained unclear. Our findings revealed that the downregulation of KIAA1199 expression can inhibit cell proliferation and motility in NSCLC cells, most probably through EGFR signaling. These findings were validated through loss‐ and gain‐of‐function experiments. We also observed an evident loss of tumor volume in mice implanted with KIAA1199‐knockdown NSCLC cells. All the results strengthened the notion that KIAA1199 is important for sustaining the aggressive phenotype of NSCLC cells.

Tumorigenesis and progression are often associated with multiple genetic changes that activate multiple signaling pathways associated with tumor cell proliferation and migration. Despite much research into the molecular pathogenesis of lung cancer, the precise mechanism by which KIAA1199 modulates NSCLC cells remains to be fully elucidated. Kateryna et al demonstrated that KIAA1199 could promote EGF‐mediated EMT process via enhancing EGFR stability in cervical cancer^24^ which arise our attention to EGFR. EGFR, as an accepted oncogene, is associated with the occurrence and development of multiple tumors^40^, ^41^ meanwhile its expression is positively related to KIAA1199. Our data also showed that downregulation of KIAA1199 leads to lower expression of pEGFR, EGFR, and several downstream signaling proteins, including EGF‐induced EMT markers. KIAA1199 not only protects EGFR from degradation but also enhances EGFR dimerization and phosphorylation of its residues. Therefore, KIAA1199 delivers phosphorylation signal to downstream kinases by directly binding to EGFR and thus controls the activation of EGFR‐mediated pathways. These findings could offer a new direction for NSCLC treatment. Epidermal growth factor receptor tyrosine kinase inhibitors (EGFR‐TKIs) are now commonly applied in advanced NSCLC with EGFR mutations. But this treatment often suffered from a limited application and secondary drug resistance,^42^ because it acts by inhibiting downstream signal transduction of EGFR, such as Erk and Akt pathways, and can only be applied to a small proportion of patients with EGFR mutation.^6^ As for many NSCLC cases without EGFR mutation, EGFR‐TKIs are unable to stop EGFR signaling from triggering activation of downstream pathways. Whereas our research showed that KIAA1199 is placed at an important junction between EGFR and other downstream kinases. Therefore, we defined KIAA1199 as a central factor connecting EGFR signaling with cell proliferation and motility in NSCLC. Hypothetically, if a novel therapy targeting KIAA1199 were to be developed, it would be effective in all NSCLC cases, with or without EGFR mutations. Interestingly, we found that Smad3 phosphorylation was also inhibited in KIAA1199‐knockdown cells. This does not contradict our finding that KIAA1199 promotes EGF‐mediated EMT but puts forward a possibility that KIAA1199 might be associated with the crosstalk between TGF‐β and EGF‐induced pathways which awaits more specific mechanistic research.

In addition to studying how KIAA1199 influences NSCLC, we also investigated the upstream regulators of KIAA1199. As is well‐known, miRNAs are associated with tumorigenesis and cancer progression by regulating key molecules.^43^, ^44^, ^45^ Mounting evidence indicates that miRNAs are crucial for the translational control of gene expression.^43^, ^46^ Since KIAA1199 was reported to interact with many miRNAs in the previous studies,^39^, ^47^, ^48^ we singled out miR‐486‐5p as a possible regulator of KIAA1199 after mining large‐scale internet databases. Moreover, Ma et al have reported that miR‐486‐5p promotes papillary thyroid cancer cell invasion by targeting KIAA1199.^49^ Therefore, we chose miR‐486‐5p as the most promising up‐stream miRNA. Our data confirmed lower miR‐486‐5p expression in NSCLC cell lines in comparison to BEAS‐2B and therefore suppressed tumor cell proliferation, migration, and invasion. Interestingly, KIAA1199 expression was proved to be down‐regulated in NSCLC cells after miR‐486‐5p overexpression. Most importantly, we presented definitive evidence that miR‐486‐5p can directly target the specific 3′‐UTR region of KIAA1199 mRNA as inferred by dual‐luciferase reporter assays. From the information above, we concluded that miR‐486‐5p regulates KIAA1199, which in turn promotes NSCLC cell proliferation and motility, completing the KIAA1199 regulatory network.

In conclusion, our research for the first time reported KIAA1199 plays a central role in connecting EGFR signaling to cell proliferation and invasion in NSCLC with or without EGFR mutations. We defined KIAA1199 as an oncogene promoting NSCLC cell proliferation and motility by transmitting EGFR signaling to Akt, Erk, and other downstream players. Herein, our data suggested a rational basis for KIAA1199 as a promising therapeutic target for NSCLC treatment.

## CONFLICT OF INTEREST

The authors declare no conflict of interest.

## AUTHOR CONTRIBUTIONS

Anqi Wang, Juan Li, and Jianjie Zhu performed majority of the experiments, analyzed data, and drafted the manuscript. Wenwen Du and Yang Zhang provided suggestions for the project and critically reviewed the paper. Tingting Cai, Ting Liu, Yulong Fu, and Yuanyuan Zeng provided us technical support. Jian‐an Huang and Zeyi Liu supervised the project and the paper. All authors read and approved the final manuscript.

## Data Availability

The data and materials supporting this study are available on reasonable request.
